# Bronchoscopic manifestations and epidemiological characteristics of lymph node fistula-type tracheobronchial tuberculosis in Hunan Province, China (2019–2023)

**DOI:** 10.1186/s12879-026-13032-z

**Published:** 2026-03-09

**Authors:** Li Luo, Lei Zhou, Linzi Luo, Quhua Yin, Zhibin Lu, Dan Feng, Qingqing Zeng, Yangbao Xiao, Jun Liang

**Affiliations:** 1 Endoscopy Center, Hunan Institute for Tuberculosis Control (Hunan Chest Hospital), Changsha, 410013 China; 2 Department of Radiology, Hunan Institute for Tuberculosis Control (Hunan Chest Hospital), Changsha, 410013 China; 3 Hunan Institute for Tuberculosis Control (Hunan Chest Hospital), Changsha, 410013 China

**Keywords:** Lymph node fistula, Tracheobronchial tuberculosis, Bronchoscopy, Elderly patients, Ulcerative perforation subtype

## Abstract

**Background:**

This study aimed to investigate the epidemiological characteristics, clinical features, bronchoscopic manifestations, and imaging findings of lymph node fistula-type tracheobronchial tuberculosis (TBTB) in Hunan Province, China, from 2019 to 2023, to provide insights for improved diagnosis and management.

**Methods:**

A retrospective analysis was conducted on 933 hospitalized cases of lymph node fistula-type TBTB in the rupture stage, identified from the bronchoscopy electronic medical record system at Hunan Chest Hospital. Patients were stratified into elderly and non-elderly groups. Comparative analyses were performed on demographics, clinical symptoms, laboratory parameters, bronchoscopic subtypes, pathogen detection, and imaging findings.

**Results:**

Patients were stratified into elderly (≥ 60 years, *n* = 424) and non-elderly (< 60 years, *n* = 509) groups. The mean patient age was 53.30 ± 18.42 years, with males slightly outnumbering females (52.09% vs. 47.91%). Elderly patients exhibited a male predominance (male-to-female ratio of 1.62:1), whereas the non-elderly group had a female predominance (male-to-female ratio of 0.57:1). Laboratory findings revealed lower hemoglobin and albumin levels but higher erythrocyte sedimentation rates (ESR) in elderly patients (*P* < 0.05 for all comparisons). Bronchoscopic examination identified ulcerative-perforative (47.91%) and granulomatous-proliferative (43.84%) as the most common fistula subtypes, with the former predominating in elderly patients (80.66%) and the latter in non-elderly patients (70.14%). Pathogen detection rates were higher in BALF samples (67.52%) than in sputum samples (57.88%), with elderly patients exhibiting higher positivity rates (*P* < 0.05). Chest CT showed mediastinal or hilar lymphadenopathy in 72.78% of cases, with partial calcification more common in elderly patients. Correlation analysis revealed that ulcerative-perforative subtypes were associated with older age, female sex, longer symptom duration, and higher ESR, while granulomatous-proliferative subtypes were linked to younger age, male sex, and better nutritional status.

**Conclusions:**

Lymph node fistula-type tracheobronchial tuberculosis (TBTB) is prevalent in elderly females, who are susceptible to severe ulcerative-perforative lesions closely associated with caseous necrosis and prolonged inflammation. Conversely, younger males mostly present with granulomatous-proliferative subtypes, suggestive of milder inflammation. Bronchoscopy and BALF testing are critical for subtype-specific diagnosis, and tailored management strategies are key to improving patient outcomes.

**Clinical trial number:**

Not applicable.

**Supplementary Information:**

The online version contains supplementary material available at 10.1186/s12879-026-13032-z.

## Background

Lymph node fistula-type tracheobronchial tuberculosis (TBTB) is a unique type of tracheobronchial tuberculosis characterized by the involvement of mediastinal or hilar lymph nodes, leading to hyperplasia, enlargement, necrosis, and eventual rupture into the trachea and bronchus. Epidemiological studies have shown varying prevalence rates of TBTB worldwide, reflecting regional differences in tuberculosis burden, healthcare access, and diagnostic capabilities [1]. In China, TBTB constitutes a significant proportion of complications in tuberculosis patients, with lymph node fistula-type TBTB (as type VI TBTB) being increasingly recognized due to advancements in diagnostic tools like bronchoscopy [2]. Globally, the prevalence of TBTB remains underreported in many developing countries, and its clinical management poses a persistent challenge [3].

Lymph node fistula-type TBTB is associated with serious clinical risks and complications. Localized ulceration of the airway fistula allows caseous necrotic material to continuously discharge into the lumen, leading to substantial bacterial load and heightened infectivity [4]. Over time, granulation tissue proliferation at the fistula site may obstruct the airway, resulting in respiratory distress. Fistulas can also encroach upon blood vessels, raising the risk of life-threatening hemoptysis [5]. Additionally, the prolonged healing process often causes structural damage to the trachea and bronchi, leading to stenosis or complete occlusion [6]. This can result in obstructive pneumonia, pulmonary atelectasis, recurrent pulmonary infections, and, in severe cases, extensive lung destruction. Such complications not only increase morbidity but also pose significant challenges for treatment and long-term recovery [7]. Addressing these risks requires early and accurate diagnosis, along with targeted therapeutic strategies.

Bronchoscopic examination plays a critical role in diagnosing and classifying lymph node fistula-type TBTB. The bronchoscopic manifestations are categorized into five subtypes: Simple Perforation, Ulcerative Perforation, Granulation Hyperplasia, Static Fistula, Scar Formation [8]. Among these, the Scar Formation subtype is a characteristic bronchoscopic finding in the late rupture stage of lymph node fistula-type TBTB. The bronchoscopic manifestations of Lymph node fistula-type TBTB in the rupture stage are classified into five subtypes: Simple Perforation Ulcerative Perforation, Granulation Hyperplasia, Static Fistula, Mixed Subtype (presenting with two or more manifestations of Simple Perforation subtype, Ulcerative Perforation subtype, Granulation Hyperplasia subtype, Static Fistula subtype).

Accurate typing is essential for understanding the disease progression and tailoring treatment strategies. With the increasing availability of bronchoscopy, the detection rate of lymph node fistula-type TBTB has significantly improved, providing clinicians with a valuable tool to assess and manage this condition [9]. However, despite these advancements, there remains a need to further analyze the clinical and bronchoscopic characteristics of this condition to aid in its early diagnosis and effective management.

This study aims to summarize the bronchoscopic manifestations and clinical characteristics of 933 patients with lymph node fistula-type TBTB treated at Hunan Chest Hospital from 2019 to 2023. By analyzing these cases, we seek to enhance clinicians’ ability to diagnose and treat this challenging condition, ultimately improving patient outcomes.

## Methods

### Study design and setting

This retrospective observational study was conducted at Hunan Provincial Chest Hospital, a tertiary referral center for tuberculosis patients in Hunan Province, China. Data were collected from the hospital’s inpatient electronic medical record system between January 1, 2019, and December 31, 2023. Patients diagnosed with tracheobronchial tuberculosis (TBTB) were identified through bronchoscopic reports. After applying the predefined inclusion and exclusion criteria, 933 patients with lymph node fistula-type TBTB in the rupture stage were enrolled for subsequent analysis.

Patients were further stratified into two age-based groups: the elderly group (≥ 60 years) and the non-elderly group (< 60 years). Clinical and bronchoscopic characteristics were compared between these groups to better understand age-related differences in lymph node fistula-type TBTB.

### Inclusion and exclusion criteria

Inclusion Criteria: Patients were diagnosed with lymph node fistula-type TBTB in accordance with the 2017 Diagnosis Standard of Pulmonary Tuberculosis (WS 288–2017) [10], the Chinese Tracheobronchial Tuberculosis Diagnosis and Treatment Guidelines (2012) [11], and other relevant literature. The following criteria were required: 1. Chest CT showing mediastinal and/or hilar lymph node enlargement with a lumen-like connection to the trachea or bronchi, indicating fistula formation; 2. Aged ≥ 12 years. Given that our hospital does not have pediatric outpatient clinics or wards and holds the qualification only for the diagnosis and treatment of adult tuberculosis patients, we mainly recruit adult patients; a small number of adolescent patients aged 12–17 years included in the cohort are complex cases referred from primary hospitals; 3. Fistula changes were confirmed by bronchoscopy, with typical findings including congested and edematous mucosa surrounding the fistula, white or grayish-white caseous necrotic material at the fistula base, and granulomatous tissue proliferation; 4. Positive laboratory results for Mycobacterium tuberculosis (MTB) in sputum or bronchoalveolar lavage fluid (BALF) based on: Acid-fast bacillus smear, nucleic acid amplification test (NAAT) for MTB, and/or positive MTB culture; 5. Histopathological evidence of tuberculous caseous necrosis from bronchoscopic biopsy.

Exclusion Criteria: 1. Patients aged < 12 years, who were excluded due to the absence of pediatric diagnosis and treatment capacity at our hospital; 2. Patients diagnosed with other subtypes of TBTB (e.g., type I: inflammatory infiltration, type II: ulcerated necrotic, type III: granulomatous proliferation, type IV: scarred stenotic, or type V: tube wall softening); 3. Lymph node fistula-type TBTB in pre-rupture or post-rupture stages (e.g., healed fistula with scar formation or pigmentation); 4. Patients with contraindications to bronchoscopy, including bleeding tendency, coagulation disorders, severe cardiopulmonary dysfunction, or refusal to undergo bronchoscopy; 5. Incomplete clinical, radiological, or laboratory data.

All procedures were conducted in accordance with the ethical standards outlined in the Declaration of Helsinki. Written informed consent for bronchoscopy was obtained from all patients. The study was approved by the Ethics Committee of Hunan Provincial Chest Hospital (Approval No. LS2021011404).

### Data collection

Comprehensive data were systematically extracted from the electronic medical records of eligible patients. Epidemiological characteristics, including age, gender, geographic origin, occupation, past medical history, history of anti-tuberculosis treatment, and comorbidities, were documented. Clinical features such as presenting symptoms (e.g., cough, sputum production, fever, hemoptysis, chest pain), symptom duration, and laboratory parameters (hemoglobin levels, albumin levels, and erythrocyte sedimentation rate) were also analyzed. Bronchoscopic findings were carefully reviewed and classified into the rupture-stage fistula subtypes (Simple Perforation, Ulcerative Perforation, Granulation Hyperplasia, Static Fistula, and Mixed Subtype). Additional bronchoscopic details, such as the location and number of fistulas and the presence of caseous necrotic material, were recorded. Besides, bronchoscopic comprehensive interventional treatment methods and the number of treatments were documented, along with anti-tuberculosis drugs and bronchoscopic fistula healing status at 2, 6, and 12 months post-interventional treatment, total duration of fistula healing (weeks from the first bronchoscopic detection of lesions to complete healing, residual pigmentation, cicatricial stenosis, or lumen occlusion), bronchial conditions at the fistula site post-treatment (pigmentation, cicatricial stenosis, lumen occlusion), and interventional treatment complications (hemorrhage, chest pain, dyspnea, sputum crust obstruction, etc.).

Microbiological data, including results from sputum and BALF tests, were evaluated using smear microscopy, NAAT, and culture. A case was defined as pathogen-positive if at least one of these tests returned a positive result. Imaging findings from chest CT scans were also reviewed to identify features such as mediastinal or hilar lymph node enlargement, calcification, lung infection, cavitation, solid changes, atelectasis, or bronchial stenosis.

Bronchoscopy and chest CT were re-examined at 6 and 12 months post-intervention to assess fistula size and lumen patency. Efficacy was graded as: (1) Marked improvement: Complete fistula healing, no significant stenosis (< 25%), and ≥ 1/2 pulmonary lesion absorption on CT. (2) Improvement: Fistula mostly closed (> 50%), lesion absorption < 2/3, and stenosis < 50%. (3) Partial improvement: Fistula mostly closed (> 25% but ≤ 50%), lesion absorption < 1/2, and stenosis < 75%. (4) Ineffective: No fistula closure, no obvious lesion absorption, and persistent stenosis/obstruction [11, 29].

### Statistical analysis

All statistical analyses were conducted using SPSS 25.0 software. Continuous variables were expressed as mean ± standard deviation (SD) for normally distributed data and as median (interquartile range, IQR) for non-normally distributed data. Categorical variables were presented as frequencies and percentages. To compare continuous variables between groups, the independent samples t-test was used for normally distributed data, while the Mann-Whitney U test was applied for non-normally distributed data. The chi-square test was used to compare categorical variables. Correlation analysis was performed using Spearman’s rank correlation coefficient to assess the relationship between age, fistula characteristics, and clinical or laboratory parameters. A two-tailed P-value of less than 0.05 was considered statistically significant.

## Results

### Patient demographics and clinical characteristics

A total of 12,991 patients diagnosed with tracheobronchial tuberculosis (TBTB) were initially identified through bronchoscopic reports. After applying the predefined inclusion and exclusion criteria, 996 cases were confirmed as lymph node fistula-type TBTB, accounting for 7.67% (996/12,991) of all TBTB cases. According to disease staging, these 996 patients were categorized into three subgroups: pre-rupture stage (1.91%, 19/996), late-rupture stage (4.42%, 44/996), and rupture stage (93.67%, 933/996). Only the 933 patients in the rupture stage were included in the subsequent analysis.

These enrolled patients had an age range of 12 to 89 years, with a mean age of 53.30 ± 18.42 years. The elderly group, defined as patients aged 60 years or older, accounted for 45.44% (424/933) of cases, while the non-elderly group, comprising patients under 60 years of age, accounted for 54.56% (509/933). Gender distribution revealed that males made up 52.09% (486/933) of the total, while 47.91% (447/933) were female. Notably, males predominated in the elderly group (male-to-female ratio of 1.62:1), whereas females were more common in the non-elderly group (male-to-female ratio of 0.57:1).Detailed age subgroup distributions and the statistically significant gender disparity between groups are summarized in Tables 1 and 2, respectively.

**Table 1:** Age distribution of patients with type VI TBTB [n (%)]

Age group	Number of cases	Ulcerative perforation subtype	Granulation hyperplasia subtype	Caseous necrosis in the lesion	Granulation hyperplasia subtype with caseous necrosis
< 20 years old	69(8.25)	0/69(0.00)	66/69(95.65)	58/69(84.06)	55/66(83.33)
≥ 20 years old,**<**30 years old	97(10.40)	6/97(6.19)	70/97(72.16)	86/97(88.66)	58/70(82.86)
≥ 30 years old, <40 years old	44(4.72)	3/44(6.82)	38/44(86.36)	35/44(79.55)	29/38(76.32)
≥ 40 years old,<50 years old	87(9.32)	11/87(12.64)	66/87(75.86)	53/87(60.92)	32/66(48.48)
≥ 50 years old,<60 years old	212(22.72)	84/212(39.62)	118/212(55.66)	131/212(61.79)	41/118(34.75)
≥ 60 years old,<70 years old	248(26.58)	193/248(77.82)	35/248(14.11)	200/248(80.64)	7/35(20.00)
≥ 70 years old,<80 years old	157(16.83)	131/157(83.44)	16/157(10.19)	137/157(87.26)	3/16(18.75)
≥ 80 years old	19(2.03)	19/19(100.00)	0/19(0.00)	19/19(100.00)	0(0.00)

*. Patients aged < 20 years: 69 cases (40 cases aged 12–17 years; 29 cases aged 18–19 years)

**Table 2:** Comparison of clinical data between Non-elderly and Elderly patients with type VI TBTB [n (%)]

Variables		Non-elderly group (*n* = 509)	Elderly group (*n* = 424)	Statistical value	*P* value
Age [years,*x*±*s*]		40.27 ± 14.90	68.94 ± 5.63	-37.46	<0.001
Gender [n (%)] Male		185(36.35)	262(61.79)	60.02	<0.001
female		324(63.65)	162(38.21)		
ESR[mm/h, M (Q1, Q3)]		28.00(12.00,50.00)	32.00(16.00,55.00)	-5.89	<0.001
Hemoglobin [mg/dl, *x*±*s*]		123.14 ± 17.41	114.83 ± 14.35	7.85	<0.001
Albumin [mg/dl, *x*±*s*]		39.49 ± 5.30	36.13 ± 4.28	10.51	<0.001
Duration of chief complaint [weeks, M (Q1, Q3)]		10.00(3.00,27.50)	12.00(4.00,35.50)	-2.88	<0.001
Symptoms [n (%)]	Cough	371(72.88)	393(92.69)	61.14	<0.001
	Coughing phlegm	288(56.58)	382(90.09)	128.34	<0.001
	Fever	63(12.38)	49(11.56)	0.15	0.70
	Hemoptysis	30(5.89)	30(7.08)	0.54	0.46
	Chest pain	53(10.41)	42(9.91)	0.07	0.80
	Shortness of breath	113(22.20)	153(36.08)	21.88	<0.001
	Night sweats	15(2.95)	18(4.25)	0.11	0.75
	Fatigue	28(5.50)	11(2.59)	0.78	0.38
	Chest tightness	48(9.43)	45(10.61)	0.36	0.55
Complications [n (%)]	Hypertension	37(7.27)	126(29.72)	80.84	<0.001
	Pneumoconiosis	49(9.63)	12(2.83)	17.49	<0.001
	Diabetes	22(4.32)	61(14.39)	28.91	<0.001
	Coronary heart disease	12(2.36)	57(13.44)	41.51	<0.001
	Arrhythmia	5(0.98)	30(7.08)	23.79	<0.001
	Slow Branch	41(8.06)	56(13.21)	6.59	0.01
	Chronic obstructive pulmonary disease	43(8.45)	73(17.22)	16.34	<0.001
	Branch expansion	16(3.14)	26(6.13)	4.81	0.03
	Pulmonary infection	184(36.15)	97(22.88)	19.36	<0.001
	Asthma	5(0.98)	10(2.36)	2.77	0.01
	Initial treatment /Re-treatment	486/23	403/21	0.10	0.76
	Drug-susceptible/ Drug-resistant	489/20	410/14	0.260	0.61

The largest occupational subgroup was farmers, followed by retirees, students, and employees (Supplementary Fig. 1). Clinical symptoms were dominated by cough (81.89%) and sputum production (71.81%), with a smaller proportion reporting shortness of breath (28.51%), fever (12.00%), and hemoptysis (6.43%). Elderly patients not only exhibited more severe symptoms but also experienced a longer duration of complaints compared to their younger counterparts. Laboratory findings further revealed that elderly patients had significantly lower hemoglobin and albumin levels and higher erythrocyte sedimentation rate (ESR) than non-elderly patients, indicating poorer nutritional status and more severe inflammation in the elderly cohort (Table 2).

### Bronchoscopic and imaging characteristics

Bronchoscopic examination revealed that the ulcerative perforation subtype was the most common in rupture-stage TBTB, and it was more prevalent in elderly patients; conversely, the granulation hyperplasia subtype was more frequent in non-elderly patients. Less common subtypes included simple perforation, static fistula, and mixed subtypes (Table 3). Bronchoscopic findings frequently revealed grayish-white caseous necrotic material at the fistula site, with congestion and edema of the surrounding mucosa.

**Table 3:** Comparison of bronchoscopic manifestations between Non-elderly and Elderly patients with type Ⅵ TBTB [n (%)]

Grouping/Project	Granulation hyperplasia subtype	Ulcerative perforation subtype	Simple perforation subtype	Static fistula subtype	Mixed subtype	Single fistula opening	Multiple fistulas (≥ 2 fistulas)	Caseous necrosisin the lesion	Location of lesion (central airway)	Location of lesion (peripheral airway)
Non-elderly group (*n* = 509)	357(70.14)	105(20.63)	25(4.91)	4(0.78)	18(3.54)	377(74.07)	132(25.93)	363(71.32)	237/663(35.76)	426/663(64.25)
Elderly group (*n* = 424)	52(12.26)	342(80.66)	0(0.00)	25(5.90)	5(1.18)	390(91.98)	34(8.02)	356(83.96)	140/475(29.47)	335/475(70.53)
*Statistical value*	337.50	333.56	32.09	20.06	/	46.45	49.74	20.93	6.63	1.80
*P value*	< 0.001	< 0.001	< 0.001	< 0.001	/	< 0.001	< 0.001	< 0.001	0.01	0.18

The mixed subtype (with two or more manifestations of granulation hyperplasia subtype, ulcerative perforation subtype, simple perforation subtype, and static fistula subtype)

A total of 1,138 fistulas were identified among the 933 patients, with most located in the peripheral airways.Single fistulas were more common than multiple fistulas, and there were significant differences in fistula number and location between the two age groups (Tables 3 and 4). Caseous necrosis was observed in 77.06% of the overall cohort, with a higher prevalence in the elderly group (Table 3).

**Table 4:** 933 Bronchoscopic fistula location in patients with type VI TBTB

Location of fistula opening	Number of cases [*n* (%)]
Protuberance	4 (0.43)
Trachea	20 (2.14)
Right main bronchus	116 (12.43)
Right upper lobe	215 (23.04)
Right middle section	142 (15.22)
Right middle lobe	162 (17.36)
Right lower lobe	75 (8.04)
Left main bronchus	95 (10.18)
Left upper lobe	109 (11.68)
Left lower lobe	119 (12.75)
Total	933

Chest CT imaging revealed mediastinal or hilar lymph node enlargement in 72.78% of patients, with partial calcification observed in 42.55%. Other findings included lung infections (44.48%), cavitation (13.61%), and lung atelectasis (9.97%). Lymph node calcification was significantly more common in elderly patients compared to the non-elderly, reflecting chronicity and disease progression in the older population. Cases with no lymph node enlargement were further stratified based on the presence or absence of calcification. Among these, 14 cases (1.50%) had calcification without lymph node enlargement, while 105 cases (11.25%) showed no calcification. This stratification aims to account for potential confounding factors, such as silicosis or pneumoconiosis, which are more prevalent among the elderly and can also lead to lymph node calcification (Table 5).

**Table 5:** Comparison of the proportion of mediastinal/hilar lymph node enlargement between Non-elderly and Elderly patients with type VI TBTB [n (%)]

Grouping/Project	No lymph node enlargement	No lymph node enlargement with calcification	No lymph node enlargement without calcification	Lymph node enlargement without calcification	Lymph node enlargement and partial calcification
Non-elderly group (*n* = 509)	119(23.38)	14(2.75)	105(20.63)	219(43.03)	171(33.60)
Elderly group (*n* = 424)	135(31.84)	24(5.66)	111(26.18)	63(14.86)	226(53.30)
*Statistical value*	8.81	1.80	1.80	129.09	36.75
*P value*	< 0.001	0.18	0.18	< 0.001	< 0.001

**Fig. 1:**
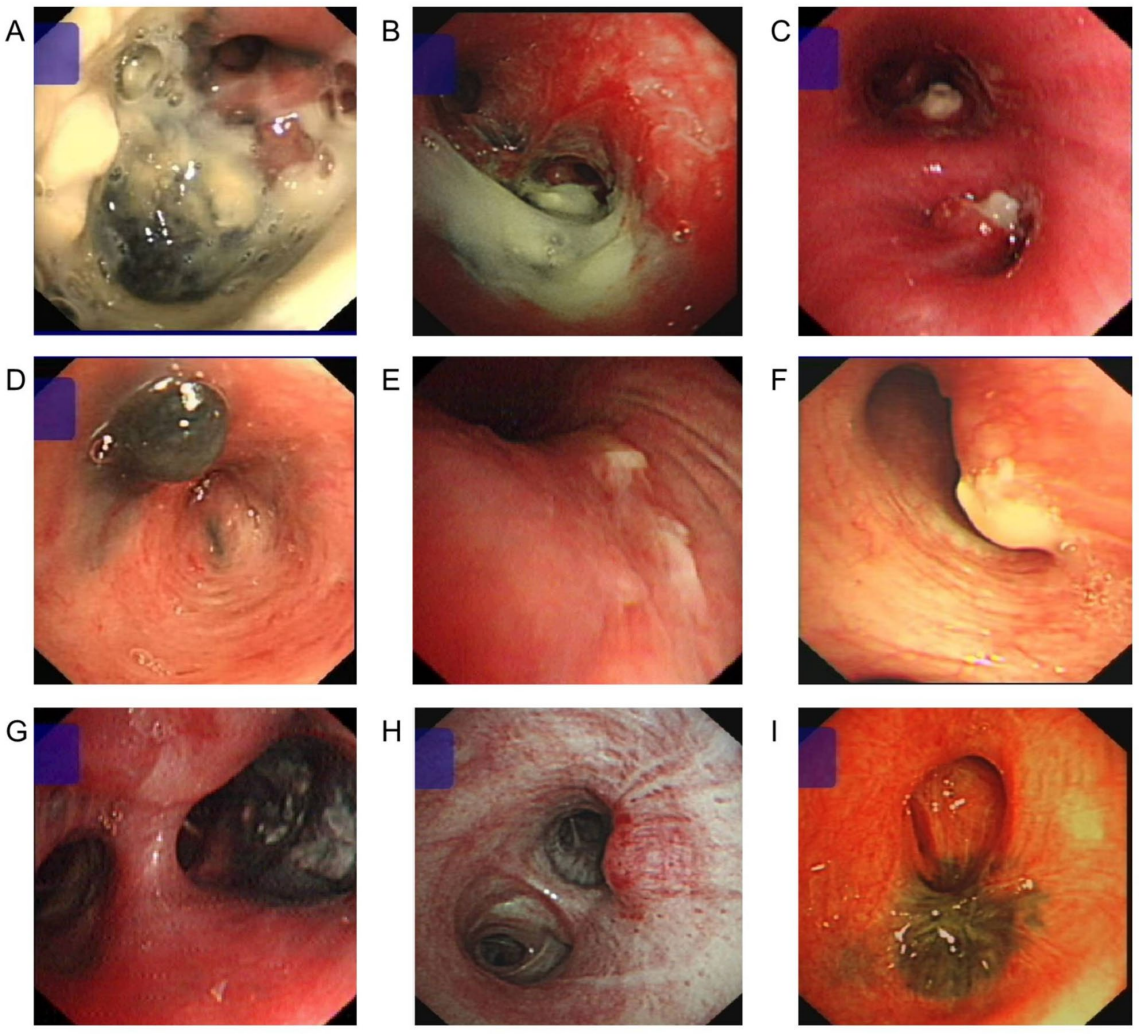
Bronchoscopic manifestations of lymph node fistula type tracheobronchial tuberculosis. **a**-**g** depict the ulceration phase, with a showing a sub-type of ulcerative perforation (patient, female, 69 years old, left lower lobe bronchial fistula with perforation, with a lot of gray-white caseous necrosis covering the surface), **b** showing a sub-type of ulcerative perforation (patient, male, 77 years old, left upper lobe bronchial fistula with deep concave, with a dark gray ulcer base and gray-white caseous necrosis covering the surface), **c** showing a sub-type of granulation hyperplasia(patient, male, 24 years old, with granulation proliferation outside the fistula in the proper and lingular lobes of the left upper lobe, wrapped in caseous necrosis), **d** showing a sub-type of granulation hyperplasia (patient, female, 47 years old, with external pressure stenosis and near occlusion of the right middle lobe lumen, with gray-black granulation in the lumen), **e** showing a sub-type of simple perforation (patient, 19 years old, with mild congestion and swelling of the local mucosa of the left main bronchus, with a small amount of white caseous necrosis in the needle-sized perforation site), **f** showing a sub-type of simple perforation (patient, female, 34 years old, with congestion and swelling of the mucosa of the left upper lobe opening, with severe external pressure stenosis of the lumen, with caseous necrosis in the perforation site), **g** showing a sub-type of static fistula (patient, male, 67 years old, with a fistula in the lateral segment of the right middle lobe that is improving but not healing, with gray-black material inside the fistula, no obvious caseous necrosis or granulation tissue); **h** shows the condition before rupture (patient male, 34 years old, local mucosal congestion in the left lower lobe opening, mild external pressure change in the lumen); **i** shows the healing of the fistula (patient, female, 65 years old, pigmentation in the opening of the right upper lobe, severe stenosis of the lumen)

Figure 1 provides representative bronchoscopic images illustrating the distinct pathological subtypes and stages of lymph node fistula-type TBTB. The ulceration phase encompassed several key subtypes (Fig. 1a and g). The ulcerative perforation subtype was characterized by mucosal ulceration and perforation, typically covered by a significant amount of gray-white caseous necrosis (Fig. 1a). A deep concave perforation with a dark gray base and overlying caseous material was also observed (Fig. 1b). The granulation hyperplasia subtype presented with prominent granulation tissue, either proliferating immediately outside the fistula and wrapped in caseous necrosis (Fig. 1c), or manifesting as gray-black granulation within the lumen, often leading to severe external pressure stenosis or near occlusion (Fig. 1d). The simple perforation subtype involved a smaller, sometimes needle-sized perforation site, usually associated with local mucosal congestion, swelling, and a small amount of caseous necrosis (Fig. 1e and f). Notably, severe external compression leading to luminal stenosis was observed even in the simple perforation subtype (Fig. 1f). Furthermore, the static fistula subtype represented a stage of improvement where the fistula was not actively healing, showing gray-black material inside without obvious caseous necrosis or granulation tissue (Fig. 1g). The phase before rupture showed localized mucosal congestion and mild changes indicative of external pressure on the lumen, preceding the eventual fistula formation (Fig. 1h). The process concluded with the healing of the fistula, which was characterized by pigmentation at the original opening site and frequently resulted in severe lumen stenosis due to scar contracture (Fig. 1i).

### Microbiological results

Pathogenetic testing showed that BALF had a higher MTB positivity rate than sputum samples, with varying positivity rates of different detection methods between the two sample types. Notably, elderly patients had higher pathogen positivity rates in both samples than non-elderly patients, indicating a higher bacterial burden in the elderly (Table 6).

**Table 6:** Comparison of pathogenic positivity rates between Non-elderly and Elderly patients with type VI TBTB [n (%)]

Grouping/Project	Sputum Total positive rate	Sputum smear	Molecular Biology	Sputum culture	BALF Total positive rate	Lavage smear	Molecular Biology	Lavage culture
Non-elderly group (*n* = 509)	214(42.04)	58(11.39)	136(26.72)	168(33.01)	282(55.40)	76(14.93)	218(42.83)	192(37.72)
Elderly group (*n* = 424)	326(76.68)	132(31.13)	234(55.19)	265(62.50)	348(82.08)	161(37.97)	287(67.69)	270(63.68)
*Statistical value*	115.19	55.56	78.35	80.91	75.04	64.80	57.57	62.35
*P value*	< 0.001	< 0.001	< 0.001	< 0.001	< 0.001	< 0.001	< 0.001	< 0.001

### Correlation analysis

Correlation analysis revealed significant associations between bronchoscopic subtypes and patient characteristics (Supplementary Table 1). The ulcerative perforation subtype was positively correlated with age, female gender, longer symptom duration, and higher ESR, while it was negatively correlated with hemoglobin and albumin levels. In contrast, the granulation hyperplasia subtype exhibited the opposite correlation trends.

Further age-stratified analysis revealed subgroup-specific patterns. In the elderly group, age showed significant opposite correlations with both subtypes; caseous necrosis strongly correlated positively with ulcerative perforation and negatively with granulation hyperplasia, whereas hemoglobin and albumin had non-significant weak correlations with ulcerative perforation (Supplementary Table 2).

In the non-elderly group, age also had significant opposite correlations with the two subtypes; caseous necrosis showed consistent but weaker trends. Notably, albumin correlated negatively with ulcerative perforation and positively with granulation hyperplasia, both statistically significant (Supplementary Table 3).

These findings suggest elderly females may be prone to severe ulcerative perforation (possibly linked to caseous necrosis), while younger males tend to have granulation hyperplasia (indicating a milder course). However, nutritional indicators (hemoglobin, albumin) lacked statistical significance in the elderly group, so their correlation with subtypes remains unclear.

### Treatment and prognosis

Among the 933 patients included in this study, 889 (95.28%) were undergoing initial treatment, while 44 (4.72%) were classified as retreatment cases. The majority of patients (899, 96.36%) were infected with drug-susceptible Mycobacterium tuberculosis, whereas 34 (3.64%) were identified as drug-resistant cases (Table 2). Non-elderly patients and elderly patients aged < 70 years received the standard anti-tuberculosis (anti-TB) regimen of 2–3HRZ/10–9HRE with a total duration of 12–18 months. In contrast, patients aged ≥ 70 years were treated with 2–3HRLfx/10–9 h for the same duration. For individuals with rifampicin intolerance, rifabutin was used as a substitute. Patients unable to tolerate pyrazinamide were prescribed levofloxacin or moxifloxacin as part of a personalized treatment regimen. Retreatment and drug-resistant cases were managed in accordance with current clinical guidelines.

Of the 933 patients with ruptured lymph node fistula-type TBTB, 754 (80.90%) received irregular bronchoscopic intervention, and 63 (6.75%) did not undergo any bronchoscopic treatment. Following informed consent, patients with the ulcerative perforation subtype were mainly treated with bronchoscopic argon plasma coagulation (APC) combined with cryotherapy plus topical isoniazid instillation, while those with the granulation hyperplasia subtype were mainly treated with bronchoscopic cryotherapy and topical isoniazid instillation.

Treatment outcomes were available for 139 elderly and 126 non-elderly patients. At 12–18 months post-treatment, 100% sputum smear conversion was achieved in both groups; the fistula healing rate was 99.14% (136/139) in the elderly and 99.21% (125/126) in the non-elderly, with a marked treatment response rate of 57.55% (80/139) and 84.13% (106/126), respectively (Supplementary Table 4).

In addition, detailed treatment data were provided by 116 patients with the ulcerative perforation subtype in the elderly group, as these patients had severe disease conditions, were the focus of attention, had a high follow-up rate, and had complete data. Their bronchoscopic intervention protocol was as follows: once a week to once every two weeks for the first 1–2 months, followed by once every two weeks to once a month for the next 3–6 months, with continuous follow-up for more than 6 months after fistula healing. Specifically, all 116 patients received bronchoscopic cryotherapy, with 106 (91.38%) undergoing additional APC. The fistula healing time was 5–76 weeks (median, 20.0 weeks; IQR, 15.0–26.0), with cumulative healing rates of 5.17% at 2 months, 74.14% at 6 months and 99.14% at 12 months (Supplementary Table 5).

No severe complications such as hemorrhage, perforation, or pneumothorax were observed during bronchoscopic interventions. Minor bleeding episodes were effectively managed with topical adrenaline or hemocoagulase. Mucus impaction following APC occurred in some cases and was successfully treated with bronchoscopic clearance when necessary.

## Discussion

Lymph node fistula-type TBTB is a distinct subtype of TBTB that presents unique clinical and bronchoscopic characteristics. According to the bronchoscopic classification, TBTB is categorized into six types: inflammatory infiltrative, ulcerated necrotic, granulomatous proliferative, scarred stenotic, softening of the ductal wall, and lymph node fistula (type VI) [11]. Among these, the lymph node fistula type, although less common, poses significant diagnostic and therapeutic challenges. Previous reports suggest that 10% to 54.3% of adult TB patients may have TBTB, with lymph node fistula-type TBTB accounting for 0.58% to 9.9% of cases [12, 13]. While most studies report these cases individually, this study, using a representative sample of 933 patients from a single tuberculosis control center in Hunan Province, China, provides a comprehensive analysis of its epidemiological, clinical, and bronchoscopic characteristics over a five-year period.

In 2024, an estimated 696,000 new tuberculosis (TB) cases were reported in China, down from 741,000 cases in 2023, corresponding to an incidence rate of 49 per 100,000 population—a 5.8% year-on-year decline. Among the 30 high TB-burden countries worldwide, China’s global ranking in new cases dropped from 3rd to 4th, accounting for 6.5% of the global total, a proportion lower than that of India (25%), Indonesia (10%), and the Philippines (6.8%) [14]. This is mainly due to tobacco use, alcohol consumption, malnutrition, diabetes mellitus, and HIV infection [15].

Regionally, the incidence rate of pulmonary TB in Hunan Province reached 56.4 per 100,000 in 2024, ranking 7th nationwide [16, 17]. This relatively high regional incidence provides a robust epidemiological foundation for conducting large-cohort studies. As a provincial tertiary grade A specialized TB hospital, we are mandated to deliver centralized treatment for refractory TB cases through a well-established three-level (provincial-municipal-county) referral network. Lymph node fistula-type tracheobronchial tuberculosis represents a particularly refractory subtype that necessitates advanced endoscopic techniques for accurate diagnosis and effective management. Our Endoscopy Center has long specialized in the diagnosis, treatment, and research of this specific TB subtype. Owing to their limited clinical capabilities, primary healthcare institutions predominantly refer such complex cases to our center.

The results of this study reveal distinct differences in the gender and age distribution of patients with lymph node fistula-type TBTB. Male patients were slightly more represented overall (male-to-female ratio of 1.09:1), but gender patterns differed by age group. Non-elderly patients (< 60 years) were predominantly female (male-to-female ratio of 0.57:1), while elderly patients (≥ 60 years) were predominantly male (male-to-female ratio of 1.62:1). These findings align with previous studies showing that adult TBTB is generally more common in females, particularly for ulcerated necrotic and scarred stenotic subtypes [2, 18]. However, the gender inversion observed in the elderly group may be attributed to biological, social, and behavioral factors. Males in older populations often experience greater exposure to TB risk factors such as smoking, alcohol consumption, and occupational hazards, which contribute to their increased susceptibility [19]. Meanwhile, females in younger populations may be more vulnerable to TBTB due to hormonal influences on immunity and higher rates of latent TB infection reactivation during pregnancy or menopause [18, 19].

The mean age of patients in this study was 53.30 ± 18.42 years. Elderly patients accounted for 45.44% of the cohort, with the majority aged 60–70 years. Compared to other subtypes of TBTB, lymph node fistula-type TBTB appears to be more common in older populations, likely due to age-related declines in immunity and a higher likelihood of reactivation of latent MTB infection. Immunosenescence, characterized by a reduction in naïve T-cell populations and impaired memory T-cell function, is a well-recognized factor contributing to TB reactivation in the elderly [20, 21]. Prolonged inflammation and weakened immune responses in older individuals may exacerbate disease severity, resulting in advanced ulcerative-perforative lesions.

The most common symptoms reported in this study were cough (81.89%) and sputum production (71.81%), which were more frequent and severe in elderly patients. Shortness of breath was also more common in the elderly, reflecting a longer duration of disease and greater airway obstruction. Laboratory findings revealed lower hemoglobin and albumin levels and higher ESR in elderly patients, indicating poorer nutritional status and heightened inflammatory responses. Hypoalbuminemia and anemia have been widely reported as markers of malnutrition and systemic inflammation in TB patients, correlating with worse clinical outcomes and more severe disease presentations [22]. These findings emphasize the need for age-specific management strategies to address the distinct clinical presentations and systemic impacts of lymph node fistula-type TBTB.

Bronchoscopy plays a pivotal role in diagnosing and classifying lymph node fistula-type TBTB [23]. In this study, the ulcerative perforation subtype was the most prevalent bronchoscopic manifestation (47.91%), particularly in elderly patients (80.66%). This subtype is characterized by deep ulcerations, mucosal perforations, and extensive caseous necrosis, which prolongs the healing process and increases the risk of complications such as airway obstruction and infection. In contrast, the granulomatous subtype, observed in 43.84% of cases, was more common in non-elderly patients (70.14%). This subtype is associated with smaller fistulas surrounded by proliferative granulation tissue, reflecting a less severe disease course.

The present findings demonstrate a positive correlation between caseous necrosis and the ulcerative perforation subtype, and a negative correlation with the granulation hyperplasia subtype. This relationship can be explained by the fact that granulation hyperplasia presents in two distinct forms: simple granulation hyperplasia (without necrosis) and necrosis-associated granulation hyperplasia. Importantly, necrosis-associated granulation hyperplasia is predominantly observed in the non-elderly population, likely reflecting heightened hypersensitivity responses in younger patients upon infection with *Mycobacterium tuberculosis*.

In contrast, elderly patients most frequently exhibit the ulcerative perforation subtype, with all such lesions invariably accompanied by caseous necrosis. This pattern is primarily attributable to a longer disease duration, age-related declines in immune function, and diminished tissue repair capacity in elderly individuals with *M. tuberculosis* infection. Middle-aged patients, who possess a moderate degree of immune competence and less pronounced hypersensitivity reactions compared to younger individuals, are more likely to present with the granulation hyperplasia subtype, and show a relatively lower incidence of concurrent necrosis.

Chest CT imaging revealed mediastinal or hilar lymph node enlargement in 72.78% of patients, with partial calcification in 42.55%. Lymph node calcification, which was more pronounced in the elderly population, is often associated with prolonged disease duration and advanced disease progression [24, 25]. This calcification reflects chronic inflammatory changes resulting from delayed resolution of mediastinal or hilar lymphadenitis and is frequently observed in patients with long-standing TB. These findings emphasize the critical need for early diagnosis and timely intervention to prevent the development of advanced structural changes in lymph nodes. However, calcification in lymph nodes is not exclusive to tuberculosis and can also result from other conditions, such as silicosis or pneumoconiosis, which are more prevalent in elderly individuals. To address this potential confounding factor, we stratified cases with no lymph node enlargement into two subgroups: those with calcification and those without calcification. This stratification provides a clearer understanding of the contributions of non-tuberculous factors to lymph node calcification and ensures a more accurate interpretation of the findings in this population.

This study highlights the importance of BALF in diagnosing lymph node fistula-type TBTB. The positivity rate for MTB was significantly higher in BALF samples (67.52%) compared to sputum samples (57.88%), with molecular biology testing demonstrating the highest sensitivity among all diagnostic methods. These findings are consistent with previous studies indicating that BALF, due to its ability to directly sample the affected airway, provides superior diagnostic yield compared to sputum in TBTB patients [23]. Elderly patients exhibited higher pathogen positivity rates, suggesting a greater bacterial burden and emphasizing the critical role of bronchoscopy in diagnosing and managing this condition. These findings underscore the need for individualized diagnostic approaches that account for patient age and disease severity.

The pathogenesis of lymph node fistula-type TBTB involves lymphatic drainage and hematogenous dissemination of MTB to hilar and mediastinal lymph nodes [26]. Caseous necrosis and rupture of these nodes lead to fistula formation, which releases infectious material into the airway [26, 27]. Correlation analysis in this study revealed that the ulcerative perforation subtype was positively associated with age, female gender, longer symptom duration, and higher ESR, while it was negatively associated with hemoglobin and albumin levels. These findings suggest that elderly patients, particularly females, are predisposed to more severe ulcerative disease due to prolonged inflammatory responses and poor nutritional status. Conversely, the granulomatous subtype was more common in younger, male patients, who generally exhibited better immune and nutritional profiles.

Combined with systemic anti-TB drug therapy, comprehensive bronchoscopic interventional therapy serves as the core strategy for the effective management of lymph node fistula-type TBTB [11, 28]. Bronchoscopic techniques, including transbronchoscopic cryotherapy, isoniazid instillation, and high-frequency electrocoagulation, have all been proven effective in eliminating caseous necrotic tissue and facilitating fistula healing [28–30]. For patients with extensive ulcerative lesions, combined rigid and flexible bronchoscopy is recommended to manage airway obstruction and reduce hemorrhage risk [31]. Stratified interventional strategies should be adopted based on bronchoscopic subtypes: the granulation hyperplasia subtype, which is characterized by multiple fistulas with repeated rupture and ulceration, can be treated with the well-established regimen of bronchoscopic cryotherapy combined with isoniazid instillation [29]; whereas the ulcerative perforation subtype, characterized by deep, large, and refractory ulcerative fistulas, requires bronchoscopic argon plasma coagulation (APC) combined with cryotherapy and isoniazid instillation to effectively remove caseous necrotic tissue and promote fistula healing [30]. After treatment, the fistula healing rates were 99.21% (125/126) in the non-elderly group (126 cases) and 99.14% (136/139) in the elderly group (139 cases), respectively. Notably, the marked effective rate was significantly higher in the non-elderly group (84.13%, 106/126) than in the elderly group (57.55%, 80/139). This therapeutic difference is attributed to the severe and irreversible damage to bronchial mucosa caused by ulcerative perforative fistulas, which often results in tracheobronchial cicatricial stenosis or even lumen occlusion post-treatment. Furthermore, elderly patients with impaired immune function and a high prevalence of comorbidities require individualized adjustment of drug dosages and prolonged treatment courses to optimize therapeutic outcomes [28, 32, 33].

### Study strengths and limitations

The strengths of this study include its large sample size, comprehensive data collection, and focus on a single TB control center, which ensures consistency in diagnostic and treatment protocols. However, the study is limited by its retrospective design and the lack of long-term follow-up data, which could provide insights into treatment outcomes and recurrence rates. Future studies should explore the molecular mechanisms underlying the different bronchoscopic subtypes and evaluate the efficacy of novel therapeutic approaches in diverse populations.

## Conclusions

Lymph node fistula-type TBTB is a severe and complex subtype of TB, which mainly affects the elderly and carries a significant morbidity risk. This study highlights the pivotal role of bronchoscopy in the diagnosis and clinical management of this disease, and emphasizes the necessity of formulating individualized treatment strategies to address the unique clinical challenges caused by age-related immune decline and poor nutritional status. Further in-depth research is required to optimize the diagnostic and therapeutic protocols, so as to improve the treatment outcomes of patients with this refractory disease.

## Supplementary Information

Below is the link to the electronic supplementary material.

Supplementary Material 1

## Data Availability

All data generated or analysed during this study are included in this published article.

## Abbreviations

TBTB: Tracheobronchial tuberculosis
TB: Tuberculosis
BALF: Bronchoalveolar lavage fluid
MTB: Mycobacterium tuberculosis
NAAT: Nucleic acid amplification test
ESR: Erythrocyte sedimentation rate
SD: Standard deviation
IQR: Interquartile range
APC: argon plasma coagulation
