# Supplementary material for: Bronchoscopic manifestations and epidemiological characteristics of lymph node fistula-type tracheobronchial tuberculosis in Hunan Province, China (2019–2023)
Source: BMC Infect Dis. 2026 Mar 9;26:772. doi: 10.1186/s12879-026-13032-z (PMC13085665; doi:10.1186/s12879-026-13032-z)
Supplement: Supplementary file 1 — Supplementary Material 1 [file 12879_2026_13032_MOESM1_ESM.docx]

**Supplementary Figure 1. Occupation information of patients (n=933).**

**Supplementary table 1. Spearman correlation analysis in total patients (n=933).**

| **Indicators** | **Age (years)** | **Gender(Male)** | **Course of illness (weeks)** | **Hemoglobin(mg/dl)** | **ESR**  **(mm/h)** | **Albumin (mg/dl)** | **Caseous necrosis in the lesion** | **Ulcerative perforation subtype** | **Granulation hyperplasia subtype** |
| --- | --- | --- | --- | --- | --- | --- | --- | --- | --- |
| **Age**  **(years) (r)** | 1.00 | -0.25** | 0.16** | -0.25** | 0.21** | -0.41** | 0.10** | 0.65** | -0.64** |
| **Gender (Male)(r)** | -0.25** | 1.00 | -0.05 | 0.37** | -0.12** | 0.07* | -0.12** | -0.29** | 0.27** |
| **Course of illness (weeks) (r)** | 0.16** | -0.05 | 1.00 | -0.04 | -0.00 | -0.07* | 0.01 | 0.12** | -0.10** |
| **Hemoglobin (mg/dl)(r)** | -0.25** | 0.37** | -0.04 | 1.00 | -0.47** | 0.52** | -0.05 | -0.20** | 0.19** |
| **ESR (mm/h)(r)** | 0.21** | -0.12** | -0.00 | -0.47** | 1.00 | -0.46** | 0.05 | 0.14** | -0.13** |
| **Albumin (mg/dl)(r)** | -0.41** | 0.07* | -0.07* | 0.52** | -0.46** | 1.00 | -0.10** | -0.30** | 0.29** |
| **Caseous necrosis in the lesion(r)** | 0.10** | -0.12** | 0.014 | -0.05 | 0.05 | -0.10** | 1.00 | 0.53** | -0.44** |
| **Ulcerative perforation subtype(r)** | 0.65** | -0.29** | 0.12** | -0.20** | 0.14** | -0.30** | 0.53** | 1.00 | -0.87** |
| **Granulation hyperplasia subtype(r)** | -0.64** | 0.27** | -0.10** | 0.19** | -0.13** | 0.29** | -0.44** | -0.87** | 1.00 |

Note: This table presents Spearman correlation coefficients (r), all retained to 2 decimal places. *P<0.05, **P<0.01 (two-tailed test).

**Supplementary table 2. Spearman correlation analysis in Elderly Group (n=424).**

|  | **Indicators** | **Age (years)** | **Gender (Male)** | **Course of illness (weeks)** | **Hemoglobin(mg/dl)** | **ESR(mm/h)** | **Albumin (mg/dl)** | **Caseous necrosis in the lesion** | **Ulcerative perforation subtype** | **Granulation hyperplasia subtype** | **Granulation hyperplasia**  **with**  **caseous necrosis** | **Granulation hyperplasia**  **without**  **caseous necrosis** |
| --- | --- | --- | --- | --- | --- | --- | --- | --- | --- | --- | --- | --- |
|  | **Age (years) (r)** | 1.00 | -0.07 | -0.04 | -0.15^**^ | 0.02 | -0.24^**^ | 0.15^**^ | 0.16^**^ | -0.13^**^ | -0.06 | -0.11^*^ |
|  | **Gender (Male) (r)** | -0.07 | 1.00 | -0.08 | 0.19^**^ | -0.01 | -0.07 | -0.20^**^ | -0.22^**^ | 0.17^**^ | 0.10^*^ | 0.00 |
|  | **Course of illness (weeks) (r)** | -0.04 | -0.08 | 1.00 | -0.10^*^ | 0.07 | -0.02 | 0.09 | 0.07 | -0.02 | 0.01 | 0.15^**^ |
|  | **Hemoglobin(mg/dl) (r)** | -0.15^**^ | 0.19^**^ | -0.10^*^ | 1.00 | -0.40^**^ | 0.47^**^ | 0.01 | -0.02 | 0.00 | 0.08 | -0.37^**^ |
|  | **ESR(mm/h) (r)** | 0.02 | -0.01 | 0.07 | -0.40^**^ | 1.00 | -0.35^**^ | 0.04 | 0.05 | -0.01 | -0.06 | 0.16^**^ |
|  | **Albumin (mg/dl) (r)** | -0.24^**^ | -0.07 | -0.02 | 0.47^**^ | -0.35^**^ | 1.00 | -0.08 | -0.09 | 0.04 | 0.05 | -0.27^**^ |
|  | **Caseous necrosis in the lesion (r)** | 0.15^**^ | -0.20^**^ | 0.09 | 0.01 | 0.04 | -0.08 | 1.00 | 0.91^**^ | -0.64^**^ | 0.07 | -0.15^**^ |
|  | **Ulcerative perforation subtype (r)** | 0.16^**^ | -0.22^**^ | 0.07 | -0.02 | 0.05 | -0.09 | 0.91^**^ | 1.00 | -0.75^**^ | -0.33^**^ | -0.13^**^ |
|  | **Granulation hyperplasia subtype (r)** | -0.13^**^ | 0.17^**^ | -0.02 | 0.00 | -0.01 | 0.04 | -0.64^**^ | -0.75^**^ | 1.00 | 0.39^**^ | 0.13^**^ |
|  | **Granulation hyperplasia**  **with**  **caseous necrosis (r)** | -0.06 | 0.10^*^ | 0.01 | 0.08 | -0.06 | 0.05 | 0.07 | -0.33^**^ | 0.39^**^ | 1.00 | -0.06 |
|  | **Granulation hyperplasia**  **without**  **caseous necrosis (r)** | -0.11^*^ | 0.00 | 0.15^**^ | -0.37^**^ | 0.16^**^ | -0.27^**^ | -0.15^**^ | -0.13^**^ | 0.13^**^ | -0.06 | 1.00 |

Note: This table presents Spearman correlation coefficients (r), all retained to 2 decimal places. *P<0.05, **P<0.01 (two-tailed test).

**Supplementary table 3. Spearman correlation analysis in Non-elderly Group (n=509).**

|  | **Indicators** | **Age (years)** | **Gender (Male)** | **Course of illness (weeks)** | **Hemoglobin(mg/dl)** | **ESR(mm/h)** | **Albumin (mg/dl)** | **Caseous necrosis in the lesion** | **Ulcerative**  **perforation subtype** | **Granulation hyperplasia subtype** | **Granulation hyperplasia**  **with**  **caseous necrosis** | **Granulation hyperplasia**  **without**  **caseous necrosis** |
| --- | --- | --- | --- | --- | --- | --- | --- | --- | --- | --- | --- | --- |
|  | **Age (years) (r)** | 1.00 | -0.06 | 0.27** | -0.01 | 0.13** | -0.26** | -0.18** | 0.42** | -0.38** | -0.48** | 0.15** |
|  | **Gender (Male) (r)** | -0.06 | 1.00 | 0.00 | 0.43** | -0.12** | 0.05 | -0.01 | -0.15** | 0.14** | 0.12* | -0.12** |
|  | **Course of illness (weeks) (r)** | 0.27** | 0.00 | 1.00 | 0.03 | -0.08 | -0.08 | -0.06 | 0.09* | -0.08 | -0.12** | 0.07 |
|  | **Hemoglobin (mg/dl) (r)** | -0.01 | 0.43** | 0.03 | 1.00 | -0.48** | 0.47** | -0.02 | -0.09* | 0.08 | 0.05 | -0.25** |
|  | **ESR (mm/h) (r)** | 0.13** | -0.12** | -0.08 | -0.48** | 1.00 | -0.48** | 0.03 | 0.03 | -0.03 | -0.00 | 0.06 |
|  | **Albumin (mg/dl) (r)** | -0.26** | 0.05 | -0.08 | 0.47** | -0.48** | 1.00 | -0.04 | -0.16** | 0.17** | 0.09* | -0.12* |
|  | **Caseous necrosis in the lesion (r)** | -0.18** | -0.01 | -0.06 | -0.02 | 0.03 | -0.04 | 1.00 | 0.34** | -0.34** | 0.58** | -0.32** |
|  | **Ulcerative perforation subtype (r)** | -0.38** | 0.14** | -0.08 | 0.08 | -0.03 | 0.17** | -0.34** | 1.00 | -0.82** | -0.42** | -0.10* |
|  | **Granulation hyperplasia subtype (r)** | 0.42** | -0.15** | 0.09* | -0.09* | 0.03 | -0.16** | 0.34** | -0.82** | 1.00 | 0.55** | 0.13** |
|  | **Granulation hyperplasia with caseous necrosis (r)** | -0.48** | 0.12* | -0.12** | 0.05 | -0.00 | 0.09* | 0.58** | -0.42** | 0.55** | 1.00 | -0.17** |
|  | **Granulation hyperplasia without caseous necrosis (r)** | 0.15** | -0.12** | 0.07 | -0.25** | 0.06 | -0.12* | -0.32** | -0.10* | 0.13** | -0.17** | 1.00 |

Note: This table presents Spearman correlation coefficients (r), all retained to 2 decimal places. *P<0.05, **P<0.01 (two-tailed test).

**Supplementary table 4. Comparison of Treatment and Prognosis between Non-elderly and Elderly Patients with Type VI TBTB (Post-Treatment).**

| **Project /Grouping** | | **Non-elderly group**  **(n=126)** | **Elderly group(n=139)** |
| --- | --- | --- | --- |
| **Age [years, M (Q1, Q3)]** | | 30.5(20.0,47.0) | 69.0(65.0,73.0) |
| **Gender [n (%)]** | **Male** | 79(62.70) | 47(33.81) |
|  | **female** | 47(37.30) | 92(66.19) |
| **Bronchoscopy treatment**  **[n (%), M (Q1, Q3)]** | **Cryotherapy** | 123(97.62),4.0(2.0,5.0) | 134(96.40),5.0(3.0,6.0) |
|  | **Argon plasma coagulation (APC)** | 4(3.17),/ | 108(77.70),3.0(2.0,4.0) |
|  | **Balloon dilation** | 4(3.17),/ | 8(5.76),/ |
|  | **Isoniazid lavage** | 126(100.00),7.0(5.0,9.0) | 139(100.00),5.0(4.0,6.0) |
| **Sputum/BALF smear conversion rate**  **[n (%)]** | **Sputum** | 126(100.00) | 139(100.00) |
|  | **BALF** | 126(100.00) | 115(99.14) |
| **Lymph Node Status on Chest CT**  **[n (%)]** | **No lymph node enlargement** | 33(26.19) | 10(7.19) |
|  | **Unchanged** | 6(4.76) | 23(16.55) |
|  | **Reduced Size** | 59(46.83) | 18(12.95) |
|  | **Reduced Size with calcification** | 28(22.22) | 88(63.31) |
| **Fistula Healing Status [n (%)]** | **Unhealed** | 1(0.79) | 1(0.72) |
|  | **Mild Healing** | 0(0.00) | 0(0.00) |
|  | **Significant Healing** | 0(0.00) | 3(0.00) |
|  | **Complete Healing** | 125(99.21 ) | 136 (99.14) |
| **Scar stenosis after treatment [n (%)]** | **No Scar stenosis** | 63(50.00) | 39(28.06) |
|  | **Mild (<25%)** | 41(32.54) | 61(43.88) |
|  | **Moderate (25–50%)** | 17(13.49) | 10(7.19) |
|  | **Severe (50–75%)** | 4(3.17) | 15(10.79) |
|  | **Total occlusion** | 1(0.79) | 14(10.07) |
| **Efficacy Evaluation [n (%)]** | **Markedly Effective** | 106(84.13) | 80 (57.55) |
|  | **Effective** | 16(12.70) | 37 (26.62) |
|  | **Partially Effective** | 3(2.38) | 21(15.11) |
|  | **Ineffective** | 1(0.79) | 1(0.72) |

**Supplementary table 5. Treatment and Prognosis of Elderly Patients with Type VI TBTB (Ulcerative Perforation Subtype, n=116).**

| **Variables/Time Point (Months)** | | **0 Months** | **2 Months** | **6 Months** | **12 Months** |
| --- | --- | --- | --- | --- | --- |
| **Bronchoscopy treatment**  **[n (%), M (Q1, Q3)]** | **Cryotherapy** | 116(100.00),5.0(4.0,6.0) | / | / | / |
|  | **Argon plasma coagulation (APC)** | 106(91.38),3.0(2.0,4.0) | / | / | / |
|  | **Balloon dilation** | 7(6.03),/ | / | / | / |
|  | **Isoniazid lavage** | 111(95.69),5.0(4.0,6.0) | / | / | / |
| **Pathogen [n (%)]** | **Sputum Total positive rate** | 101(87.07) | 19(16.38) | 1(0.86) | 0(0.00) |
|  | **Sputum smear** | 44(38.26) | 1(0.87) | 0(0.00) | 0(0.00) |
|  | **Molecular Biology** | 79(68.10) | 3(2.61) | 0(0.00) | 0(0.00) |
|  | **Sputum culture** | 88(76.52) | 18(15.65) | 1(0.86) | 0(0.00) |
|  | **BALF Total positive rate** | 110(94.83) | 49(42.24) | 2(1.74) | 1(0.86) |
|  | **Lavage smear** | 62(53.91) | 2(1.74) | 0(0.00) | 0(0.00) |
|  | **Molecular Biology** | 97(83.62) | 24(20.87) | 2(1.74) | 1(0.86) |
|  | **Lavage culture** | 89(77.39) | 33(28.69) | 0(0.00) | 0(0.00) |
| **Lymph Node Status on Chest CT [n (%)]** | **No lymph node enlargement** | 7(6.03) | 7(6.03) | 7(6.03) | 7(6.03) |
|  | **Unchanged** | / | 61(52.59) | 27(23.28) | 19(16.38) |
|  | **Reduced Size** | / | 7(6.03) | 12(10.34) | 14(12.07) |
|  | **Reduced Size With calcification** | 109(93.97) | 41(35.34) | 70(60.34) | 76(65.52) |
| **Fistula Healing Status [n (%)]** | **Unhealed** | 116(100.00) | 16(13.79) | 1 (0.86) | 0(0.00) |
|  | **Mild Healing** | 0(0.00) | 47(40.52) | 24 (20.69) | 0(0.00) |
|  | **Significant Healing** | 0(0.00) | 46(39.66) | 24 (20.69) | 0(0.00) |
|  | **Complete Healing** | 0(0.00) | 6(5.17) | 86(74.14) | 115(99.14) |
| **Scar stenosis after treatment [n (%)]** | **Mild (<25%)** | / | / | / | 50(43.10) |
|  | **Moderate (25–50%)** | / | / | / | 11(9.48) |
|  | **Severe (50–75%)** | / | / | / | 18(15.52) |
|  | **Total occlusion** | / | / | / | 8(6.90) |
| **Efficacy Evaluation [n (%)]** | **Markedly Effective** | / | / | 55(47.41) | 62(53.45) |
|  | **Effective** | / | / | 38(32.76) | 34(29.31) |
|  | **Partially Effective** | / | / | 22(18.97) | 19(16.38) |
|  | **Ineffective** | / | / | 1(0.86) | 1(0.86) |
